# Community-acquired pneumonia caused by methicillin-resistant *Staphylococcus aureus* in a Chinese adult: A case report

**DOI:** 10.1097/MD.0000000000020914

**Published:** 2020-06-26

**Authors:** Huan Xia, Jinying Gao, Ming Xiu, Dan Li

**Affiliations:** a Department of Respiratory Medicine; b Department of Intensive Care Unit Group One, The First Hospital of Jilin University, Changchun, Jilin, China.

**Keywords:** community-acquired pneumonia, methicillin-resistant *Staphylococcus aureus*, Panton–Valentine leukocidin

## Abstract

**Rationale:**

Methicillin-resistant *Staphylococcus aureus* (MRSA) has been established as an important cause of severe community-acquired pneumonia (CAP) with very high mortality. Panton–Valentine leukocidin (PVL) producing MRSA has been reported to be associated with necrotizing pneumonia and worse outcome. The incidence of community-acquired MRSA (CA-MRSA) pneumonia is very low, as only a few CA-MRSA pneumonia cases were reported in the last few years. We present a case of severe CAP caused by PVL-positive MRSA with ensuing septic shock.

**Patient concerns:**

A 68-year-old male with no concerning medical history had developed a fever that reached 39.0°C, a productive cough that was sustained for 5 days, and hypodynamia. He was treated with azithromycin and alexipyretic in a nearby clinic for 2 days in which the symptoms were alleviated. However, 1 day later, the symptoms worsened, and he was taken to a local Chinese medicine hospital for traditional medicine treatment. However, his clinical condition deteriorated rapidly, and he then developed dyspnea and hemoptysis.

**Diagnosis:**

CA-MRSA pneumonia and septic shock. The sputum culture showed MRSA. Polymerase chain reaction of MRSA isolates was positive for PVL genes.

**Interventions:**

Mechanical ventilation, fluid resuscitation, and antibiotic therapy were performed. Antibiotic therapy included mezlocillin sodium/sulbactam sodium, linezolid, and oseltamivir.

**Outcomes:**

He died after 12 hours of treatment.

**Lessons:**

This is a report of severe pneumonia due to PVL-positive CA-MRSA in a healthy adult. CA-MRSA should be considered a pathogen of severe CAP, especially when combined with septic shock in previously healthy individuals.

## 1 Introduction

Although methicillin-resistant *Staphylococcus aureus* (MRSA) has been known to be associated with nosocomial pneumonia, several reports have also described cases of community-acquired pneumonia (CAP) that was caused by MRSA. The estimated incidence of community-acquired MRSA (CA-MRSA) pneumonia is 0.51 to 0.64 cases per 100,000.^[1]^ An international multicenter study indicated that the overall prevalence of confirmed MRSA CAP was 3% among 3193 CAP patients with microbiology testing, ranging from 2.4% in Europe to 5.4% in South America.^[2]^ MRSA can cause severe CAP, which leads to critical illness and sometimes death. Self and colleagues observed that chronic hemodialysis use was more common among patients with MRSA than pneumococcal and all-cause non-*S aureus* CAP, whereas other clinical features at admission were similar.^[3]^ Patients with CA-MRSA pneumonia had more severe clinical outcomes than those with pneumococcal CAP, including intensive care unit admission and in-patient mortality.^[3]^ Some research reported the mortality of CA-MRSA pneumonia is as high as 56% to 63%.^[4,5]^ Postinfluenza bacterial pneumonia is a leading cause of influenza-associated death and CA-MRSA pneumonia has been reported to occur following influenza infection, posing a therapeutic challenge because of the high risk of inappropriate empiric antimicrobial therapy and poor clinical outcomes.^[6,7]^ The Panton-Valentine leukocidin (PVL) virulence is typically associated with CA-MRSA strains.^[8]^ CAP due to MRSA carrying the PVL gene can perform as extensive lung necrosis, multilobular infiltrates, leucopenia, hemoptysis, and sepsis, leading to a higher lethality rate.^[9,10]^

Herein, we present a case of severe CAP caused by MRSA with septic shock in a 68-year-old previously healthy male. We also review the literature related to CA-MRSA pneumonia.

## 2 Case presentation

**Figure 1:**
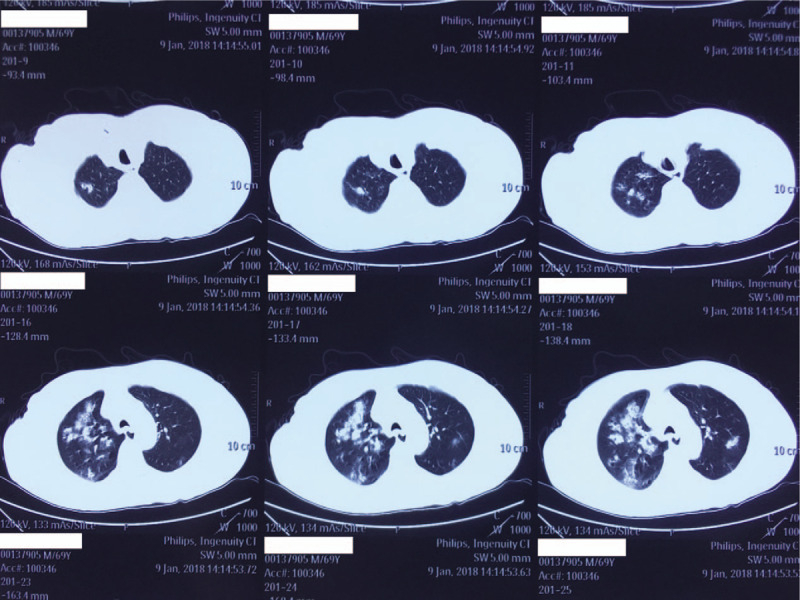
A computed tomography scan of the chest performed on January 9, 2018.

**Figure 2:**
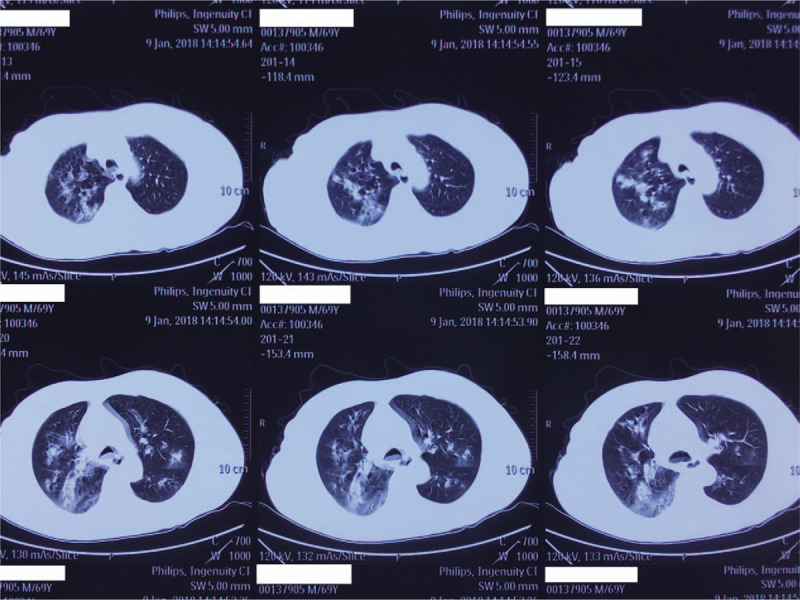
A computed tomography scan of the chest performed on January 9, 2018.

**Figure 3:**
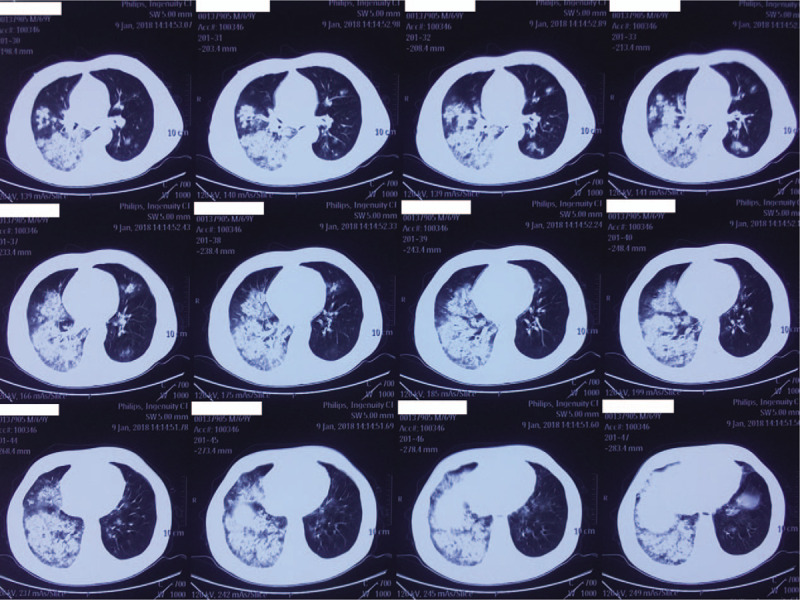
A computed tomography scan of the chest performed on January 9, 2018.

A 68-year-old male with no concerning medical history had developed a fever that reached 39.0°C, a productive cough that was sustained for 5 days and hypodynamia. He went to a nearby clinic and he was treated with azithromycin and alexipyretic for 2 days, in which the symptoms alleviated. However, 1 day later, the symptoms worsened, and he was taken to local Chinese medicine hospital for traditional medicine treatment. However, his clinical condition deteriorated rapidly, and he then developed dyspnea and hemoptysis. He was then taken to another hospital for a chest computed tomography (CT) scan, which showed consolidations with interspersed small lucent areas in both lungs (Figs. 1–3). Following this, he came to our emergency department and was intubated for mechanical ventilation. He was then admitted to our respiratory intensive care unit (RICU).

He had an appendectomy 10 years ago, but apart from that he had no other significant medical history. He also had a 15-pack-year smoking history and a long-term history of alcohol intake with an average of 100 g/day.

His vital signs on admission to our RICU revealed a heart rate of 106 beats/min and a blood pressure reading of 70/52 mmHg with a shock manifestation. The oxygen saturation was 83% under ambu bag operation through a trachea cannula. Auscultation revealed lower respiratory sounds and a few moist rales in the base areas of both lungs. There were no wounds on his body, arms, or legs.

Initial blood investigations showed a white blood cell count (WBC) of 1.2 × 10^9^ cells/L made up of 75% neutrophils, a lymphocyte count of 0.21 × 10^9^ cells/L and platelet count of 94 × 10^9^ cells/L. Arterial blood gas analysis showed a pH of 7.19, a partial pressure of carbon dioxide (PaCO_2_) at 39 mmHg, a partial pressure of oxygen (PO_2_) at 57 mmHg, and a lactate level at 5.6 mmol/L. C-reactive protein and procalcitonin were markedly elevated at 61.24 mg/L and >400 ng/mL, respectively. He appeared to have hypoproteinemia as the total protein level was 42.2 g/L and the albumin level was 22.8 g/L. Renal function was also poor as the blood urea nitrogen level was 12.62 mmol/L and the creatinine level was 231.2 mmol/L. Blood coagulation function showed activated partial thromboplastin time at 41.5 seconds, plasma prothrombin time at 14.9 seconds, the international normalized ratio was 1.27, and prothrombin activity was at 65%. The level of blood calcium was as low as 1.67 mmol/L. The plasma concentration of 1,3 beta-d-glucan was 10 ng/mL. The erythrocyte sedimentation rate was normal. Plasma d-dimer and fibrinogen degradation product was 2708 μg/L and 15.6 μg/ml, respectively. B-type natriuretic peptide was 5690 pg/mL. Myohemoglobin was 6633 ng/mL and CK-MB was 7.72 ng/mL. The routine urine test showed bilirubin 1+, ketone 1+, nitrite +, and WBC 30.1 cells/μL.

He had received mechanical ventilation immediately with FiO_2_ at 1.0 and positive-expiratory pressure at 10 cmH_2_O. He was then sedated, and a neuromuscular blockade was given to him. Noradrenaline was intravenously injected and fluid resuscitation was conducted. Antibiotic therapy was also conducted and included mezlocillin sodium/sulbactam sodium, linezolid, and oseltamivir. Hydrocortisone was administrated due to septic shock. His airway had then started to fill with bloody secretion. Although he was given immediate therapy, the condition of his hypoxemia did not improve and extracorporeal membrane oxygenation (ECMO) was advised. The patient's family members refused ECMO treatment for financial reasons. During his second day of hospitalization, the patient died.

On day 4, a culture of sputum showed MRSA that was resistant to oxacillin, benzylpenicillin, erythromycin, and clindamycin, but susceptible to ciprofloxacin, vancomycin, levofloxacin, quinupristin/dalfopri, oxacillin, moxifloxcin, rifampicin, trimethoprim/sulfamet, tigecyclin, tetracycline, and gentamicin.

Polymerase chain reaction (PCR) of MRSA isolates obtained from the patient's sputum was conducted as described in previous reports.^[11]^ The results were positive for PVL genes.

Informed written consent was obtained from the son of the patient for publication of this case report and accompanying images.

## 3 Discussion

MRSA has been recognized as a cause of severe CAP though not common.^[12]^ CA-MRSA pneumonia usually occurs in young otherwise healthy individuals and can progress rapidly with various complications leading to a high mortality rate.^[13]^ Some studies have defined infections with CA-MRSA as follows: an MRSA infection identified within 48 hours of admission to a hospital; no history of hospitalization, surgery, dialysis, or residence in a long-term care facility within 1 year of the MRSA culture date; without a permanent indwelling catheter or percutaneous medical device present at the time of culture; and without a known previous MRSA infection or colonization before the study period.^[14–16]^ Our patient was previously healthy but then developed a fever, productive cough, dyspnea, and leucopenia. His chest CT scans showed multilobular infiltrates and consolidations in both lungs. His sputum yielded strains of MRSA. Therefore, we defined the patient as having CA-MRSA pneumonia.

The PVL is a pore-forming toxin secreted by MRSA and it has been proven to induce rapid activation and cell death in human neutrophils, thus playing an important role in the development and outcome of CAP.^[17]^ A report by Bhatta et al showed that 90.4% CA-MRSA were PVL-positive, whereas only 7.1% hospital-associated MRSA were PVL-positive, suggesting that PVL may be a marker of CA-MRSA.^[8]^ It has also been reported that CAP caused by MRSA carrying the PVL gene can also lead to extensive lung necrosis, multilobular infiltrates, leucopenia, hemoptysis, and sepsis.^[9]^ The presentation of the patient we described was consistent with the production of PVL, which was later confirmed by PCR.^[11]^ The patient we described showed leucopenia, hemoptysis, and sepsis shock which were typical of PVL-positive MRSA pneumonia. Necrotizing pneumonia may the following features multiple pulmonary consolidations and necrosis of the lung tissue, which can result in cavitations and collection of pus in the pleural cavity.^[18]^ However, not all cases are characterized by pulmonary cavitation. The radiological findings of our patient included multilobular infiltrates and pulmonary consolidations; however, there were no typical cavities on his chest CT scans.

In recent years, some cases of severe CA-MRSA pneumonia have been reported to be associated with previously having influenza,^[5,6,13,19]^ raising concerns about PVL-MRSA pneumonia during influenza epidemics. Viral and other types of infection may damage epithelial cells in the airway, leading to the migration of PVL-positive MRSA to the basement membrane.^[20]^ PVL could then cause pulmonary necrosis through tissue destruction via infiltration by neutrophils and macrophages. Our patient presented with a fever, a productive cough, and fatigue, but did not present with a sore throat, nasal congestion, running nose, or headache. Onset of symptoms from this patient was within the influenza season; thus, we prescribed oseltamivir. Unfortunately, the patient died the next morning; therefore, a test of the influenza virus nucleic acid PCR was not conducted.

It has been reported that CA-MRSA is typically susceptible to most non-β-lactam antibiotics and HA-MRSA exhibits resistance to numerous agents. However, increasing non-β-lactam resistance of MRSA has been found in recent years varying between countries and clones.^[21]^ The MRSA grown from the patient's sputum was resistant to erythromycin and clindamycin, with exceptions being to oxacillin and benzylpenicillin.

Although the prevalence of CA-MRSA pneumonia was low, 29.8% of hospitalized CAP patients still received empirical anti-MRSA antibiotics and only 0.7% of cases were CA-MRSA pneumonia.^[3]^ It is vital to understand that CA-MRSA can cause lethal pneumonia, even in previously healthy persons. Therefore, early recognition of this infection and timely antimicrobial therapy are important to improve the prognosis. Empirical therapy for MRSA is only recommended for hospitalized patients with severe CAP defined by any one of the following: ①a requirement for intensive care unit admission, ②necrotizing or cavitary infiltrates, or ③emphysema, pending sputum and/or blood culture results.^[22]^ Vancomycin and linezolid were recommended as first-line agents for CA-MRSA infection.^[23]^ Our patient was initially given linezolid. However, this critically ill patient died after 12 hours.

In conclusion, we reported a severe CA-MRSA pneumonia along with septic shock in a patient without any concerning medical history. We should consider the possibility of a CA-MRSA infection in cases with severe CAP and necrotizing pneumonia, especially with the possibility of developing septic shock.

## Author contributions

**Formal analysis:** Huan Xia, Jinying Gao, Ming Xiu

**Investigation:** Jinying Gao and Ming Xiu

**Writing – original draft:** Huan Xia

**Writing – review & editing:** Dan Li
